# How Are They Gonna Look at Me? A Constructivist Study of Medical Students’ Categorisation and its Impact on Study Experiences

**DOI:** 10.5334/pme.2249

**Published:** 2026-08-26

**Authors:** Albertine Zanting, Janneke Frambach, Anja Krumeich, Agnes Meershoek

**Affiliations:** 1 Department of Health, Ethics and Society, School of Health Professions Education, Faculty of Health, Medicine and Life Sciences, Maastricht University, Maastricht, The Netherlands; 2 Department of Educational Development and Research, School of Health Professions Education, Faculty of Health, Medicine and Life Sciences, Maastricht University, Maastricht, The Netherlands; 3 Department of Health, Ethics and Society, School Caphri, Care and Public Health Research Institute, School of Health Professions Education, Faculty of Health, Medicine and Life Sciences, Maastricht University, Maastricht, The Netherlands; 4 Department of Health, Ethics and Society, School Caphri, Care and Public Health Research Institute, Faculty of Health, Medicine and Life Sciences, Maastricht University, Maastricht, The Netherlands

## Abstract

**Introduction:**

Research shows that medical students from certain minoritised backgrounds often face discrimination and reduced belonging, harming wellbeing and performance. However, such research rarely justifies its focus on these backgrounds, taking the selected categories of minoritized students for granted, even though categorisation itself is neither neutral nor harmless. To offer an alternative perspective, we used theoretical perspectives of categorisation to examine categories students construct for themselves and others, what meanings they attribute to these constructions, and how this affects their study experiences.

**Method:**

Using a constructivist approach, we conducted semi-structured interviews with 11 students from two Dutch medical programmes. Students reflected on experiences from application to clinical rotations. Transcripts were coded using theoretical concepts of categorisation. The analysis focused on how and when students invoked categories, their attached meanings, and impact. Findings were synthesized into three crafted stories illustrating students’ experiences.

**Results:**

Students described categorising themselves and others fluidly, shifting across time and context. Categories became salient when students deviated from an implicit norm (e.g., identifying as lesbian in heteronormative environments or Black among white peers). Students and others valued each other positively, yet sometimes overemphasized categories and negatives. Such dynamics shaped feelings of belonging and exclusion and, sometimes, contributed to dropout.

**Discussion:**

Improving students’ learning experiences requires acknowledging categorisation’s omnipresent nature and its interconnected dynamics. Focusing on individuals’ experiences and implicit norms – rather than standard categories – in all stages of educational practice and research is essential for fostering diversity and inclusion in the learning and healthcare environment.

## Introduction

Despite increasing recognition that diversity in the healthcare workforce promotes more equitable care [1,2,3], significant gaps in representation persist. Research indicates that students from specific groups often face unequal treatment and distinct challenges that negatively impact their academic and personal lives. These include limited access to role models and medical network [4], insufficient mentorship [1,5], racial, ethnic, gender and social disparities, microaggressions, stereotyping, discrimination [2,3,5,6,7,8,9,10], a lack of safe climate [8,11], prejudices, stigmas and implicit biases [12,13], and bias in clinical assessments [2]. Such experiences diminish students’ sense of belonging [4,8], increase emotional stress [6,14], and adversely affect academic performance and professional development [2,4,7,11,12].

Most existing studies link these experiences to specific groups—such as ethnic minorities, migrants, underrepresented or transgender students—but rarely examine how or why these categories are being referred to. Often, such categories are replicated uncritically from previous studies, the categories used are limited and seem to be taken for granted. Yet categorisation itself, understood as the act or process of characterising and making distinctions between people, is neither neutral nor harmless and seriously impacts people concerned. In broader health and social sciences, it is acknowledged that categories are socially constructed and have real-world consequences [15,16,17]. For instance, Helberg-Proctor (2017) notes that racial and ethnic categories persist due to their institutionalisation in research practices, shaping public health priorities and interventions in ways that may stigmatise certain groups [18]. This same logic applies to education: unconsciously and repeatedly categorizing students may reinforce exclusion and seriously influence students’ learning experiences and study life. If unexamined, medical education researchers, educators and supervisors risk staying uninformed about the impact of categorisation, leading to continued stigmatisation and negative learning experiences of minoritised future doctors. More insights in the everyday educational phenomenon of categorisation may result in a more equitable admission process, less dropout, enhanced medical knowledge, and better qualified doctors.

Theories of categorisation, such as (but not limited to) othering and social identity, have highlighted the complexity of creating categories through different interacting and interinfluencing dynamics. Firstly, while categories are often presented as dichotomies—majority vs. minority, us vs. them— this presentation is one-dimensional and disregards that people may simultaneously identify with multiple groups or switch affiliations depending on time and setting [19,20,21,22]. Secondly, by drawing boundaries between groups and placing others outside those boundaries we feel where we belong and we exclude others, and we establish our identities [22,23]. This phenomenon of inclusion and exclusion is inherently paradoxical: people want both full acceptance by and belonging to a larger social unit while at the same time being able to maintain one’s distinctiveness and uniqueness. Simultaneous compliance with both aims is impossible without losing benefits or rights available to other members of the social unit [24]. Thirdly, categories can be self-assigned (avowed) or externally imposed (ascribed): people categorise both others as well as themselves, and these categorisations may not be alike [20]. Fourth, categories carry (usually implied) meanings and implications, which may be negative and positive, as well as norm setting and hierarchically interrelated. Two examples: strong leadership is associated by some with masculinity, which creates gender hierarchy [25], and some argue that the ‘norm’ in science, medicine and health is ‘white’ or ‘European’, resulting in disparities in health care [19]. Fifth, deviations from an implicit norm marginalise those who do not conform, resulting in people experiencing both being categorised too much (stereotyped, stigmatised), but also not being sufficiently recognised for who they are [21]. In sum, categorisation is a paradoxical, multidirectional, and meaning bearing phenomenon with categories acting as fluid occasional labels. These dynamics underscore that categorisation is deeply political [26], shaping not only how people are perceived but also how they engage in their educational environment.

In this study, we draw on these theoretical perspectives to explore how medical students experience categorisation in their everyday academic lives. We examine three research questions: 1) how (and what) categories are dynamically constructed by students themselves and by others in their educational environment (as perceived by these students), 2) what meanings are attributed to these constructions, and 3) how such categorisation affects students’ learning experiences and study life.

By unpacking the nuanced processes of categorisation, our study aims to inform education policy and practice focused on students’ study experiences. A deeper understanding of how student differences are constructed can help create more inclusive environments, contributing to a healthcare workforce that better reflects and serves diverse populations.

## Methodology

### Research approach

In line with the constructivist paradigm, we explored how students narrate their practices of categorisation – of themselves as well as of others – and how these practices impacted their learning experiences, with the aim of increasing the understanding of categorisation [27]. Using the qualitative concept of ‘thick description’, we sought to describe and interpret social actions within a particular context, to capture the thoughts and experiences of participants, and to assign purpose and intentionality to these actions [28]. To achieve this, we used ‘composite narratives’ as the method to communicate our findings. The benefits of composite narratives include emphasising participant voices, conveying research rigour, presenting multifaceted ideas succinctly, encouraging reader resonance, and protecting participant anonymity [29].

### Research setting

Focusing on a site that provides potentially broad information on student categorisation, we recruited students from a medical school offering programmes in several languages to students coming from different countries. This Dutch school offers a six-year medical programme, divided into a three-year bachelor’s and three-year master’s phase. The bachelor’s programme is offered in both Dutch and English. The master’s programme is offered only in Dutch, due to the extensive patient contacts during rotations. The medical school employs the Problem Based Learning (PBL) educational method.

### Selection of participants

Given the exploratory nature of this study and our research paradigm, we included students with a broad variety of experiences and backgrounds from all medical programmes offered at the site. Students were recruited via newsletters and through ‘snowballing’. We used the concept of ‘information power’ and its five relevant aspects to consider [30] to determine the final number of participants. Because of the broad (and not narrow) aim of the study, the dense (instead of sparse) specificity of the experiences and knowledge of the participants, the use of extensive (and not limited) theory, the strong and clear (rather than weak) interview dialogue, and the exploratory (instead of in-depth) analysis strategy [30], eleven students ultimately participated in this study. Three were from the bachelor/preclinical years, and eight from the master’s/clinical years. Four participants were following the International Track in Medicine and seven were from the Dutch Track. Nine students were currently enrolled in the medical course, while two had taken a year-long break to complete a master’s in another programme at the same university.

### Data collection

Through eleven semi-structured online interviews and one focus group session, we collected narratives about the students’ experiences throughout their student lives. While the interviews aimed to document participants’ individual experiences and stories, the focus group session allowed us to capture participants’ reactions to each other’s experiences and perspectives, thereby identifying similarities and differences in viewpoints, and enriching the data set [31]. Both data collection methods (twelve transcripts in total) contributed to the analytic process. The interview and focus group sessions lasted between 40 and 80 minutes. An interview guide with open ended questions followed the stages of a student’s study life: information gathering, selection and admission, introduction, during studies (e.g. classroom interaction), during rotations, and expectations for the future. In line with the study’s aim to explore categorisation in practice, participants were invited to talk about their experiences during these stages without explicitly referring to predefined categories. The audio-taped interviews and focus group session were conducted by two research assistants, whose similarity to the interviewees in terms of age and being former (medical) students facilitated trust.

### Data analysis

All interviews and the focus group were transcribed verbatim. One researcher (AZ) inductively coded the transcripts, focusing on references (or the absence thereof) to characterisations of students or others, the meaning attached to these characterisations, by whom they were made and in which context, and what that meant for students in terms of learning experiences and interaction during their studies. Using the aforementioned theories of categorisation as sensitising concepts, the research team (AZ, AK, AM, JF) analysed the materials in several rounds of familiarisation, discussions and pattern-seeking. In this process, which allowed them to continuously reflect on their biases, the research team began crafting stories based on the data, in line with the ‘composite narratives’ approach. As Crowther explains: ‘crafting stories from transcript data is about bringing the story together in a way that “shows” what the researcher is noticing and interpreting while working with the data’ [32]. The crafting process highlights the phenomenon in a more concise and readable format, reducing the need for lengthy verbatim data, while staying close to students’ concrete daily life experiences [32]. Three stories were constructed from the entire data set as illustrations of the research findings, which allowed the research team to show the multilayeredness of categorisation and include common patterns as well as diverse and divergent perspectives across the data set. The stories were composed by weaving together quotes (in italics) from interviewed students, complemented with summarized experiences in regular font. One researcher (AZ) crafted the stories, which were then re-edited in discussions with the other research team members (AK, AM and JF) as the analysis deepened. Crafted stories proved to be an appropriate way to report the findings and to demonstrate how the dynamic interrelatedness of categorisation converges in one person [33,34]. Details of nationality were altered to ensure anonymity.

### Ethical considerations

Ethical clearance was obtained from the Global Health Program Ethical Review Committee of Maastricht University under registration number FHML/GH_2020–2021.064. Data were analysed, stored, and reported pseudonymously. Prior to the interview, participants provided informed consent. During and after the interviews, they were given the opportunity to omit information.

### Reflexivity

By consciously questioning our positionality within the research team and towards the data, the team contributed to trustworthiness [35]. Our interpretations of the data were shaped by our varied academic training, our backgrounds as social scientists using constructivist approaches to research from a critical perspective, and our different experiences and observations of cultural diversity education as teachers in medical and health professions programmes. We share – and differ in – lived experiences of cultural diversity in our professional and personal lives, and we continuously sought to remain aware of the ways in which our backgrounds shaped our views. Two research assistants with different backgrounds were involved in data collection, which we believe enriched the data. We selected a data reporting strategy (crafted stories) that explicitly reflects the participants’ voices. We added our own interpretations after the stories, reflecting our voices as researchers and individuals, yet guided by theoretical insights that we made transparent.

## Results

To clarify the several interinfluencing dynamics of categorisation, we prepared three fictitious stories consisting of compilations of the concrete experiences of all eleven participants, crafted from different parts of the verbatim transcribed interviews. The narratives demonstrate the complex dynamics of categorisation, which we explained by adding our reflections after the crafted stories. We chose different types of flowers as fictitious student names, as we felt these are neutral as well as personal.

### Student 1: Dahlia

*I’m 22* […] *I am half* [French], *half Dutch*. (B13) After my international high school experiences and smooth selection process, I easily integrated in the English programme and student life. *[…]* I love PBL, *cause there are some people who are fast learners and others who learn more slow* (B13). *And I love that we’re all very different, like from different ages and […] countries (*B17)

*I’m not from a very privileged background, you know. I saw my parents struggling. [*…*] And […] my fellow students, […] I can imagine like them growing up in the Netherlands in these very […] white, privileged families*. (B12) Being conscious about my privileges as a white person, I raise my voice when in the course material or classroom discussion certain terms are being used. *For example, [in some cases] in Dutch they have a tendency to say negroide or negroid, and yeah, that kinda makes me uncomfortable* (B02). But other students *just use those words […], they don’t really see anything behind it or are oblivious to it. (B02)*

For social contacts, *I googled what churches there were in Maastricht. […] we very quickly become sort of like a family and it’s really nice […]*. (B17)

Luckily, the interaction with Dutch peers is easier during the master than during the bachelor. *‘As a coassistent [intern], it’s really nice to have another student that’s also in similar struggle as you, so you’re able to interact and relate more […]. But it’s very different from person to person*. (B01)

The only thing that keeps bothering me are discussions with colleagues who assume that *if you have faith then you’re a big dumb really like: how could you believe in such a thing when science explains everything when actually, they’ve never heard our perspective in which religion and science really don’t contradict each other. (B17)*.

Overall, I enjoy my studies and I am confident about future medical training.

### Student 2: Crocus

I am a 6th year student from one of the Dutch Antilles. *I have the Dutch nationality so things were a bit easier for me when applying (B01). Then when I moved here and saw the white majority, it was definitely shocking. […] I was the only person of color* (B01). *I think for them* [Dutch classmates]*, it wasn’t really an issue. But for me, I was kind of basically all the time, the only black person there from* [the Antilles]. (B16). *I haven’t had experiences where teachers […] would or seemed to expect less from me or think that I’m performing at a lower standard* (B14), coming from the Dutch Antilles.

*The majority of medical students are women and at the introduction day they used to talk about topics that I really didn’t care about – so I couldn’t really join the discussion* (B02).

*In my first year, I tried to* [have] *only Dutch friends, but that really backfired […]. There’s just some parts of the culture which I cannot bring myself to acclimatise to*. (B06).

I reached out to my mentor, who was of great help, as well as peers at the Caribbean students’ event. I became more aware of my culture shock. *In general, with Dutch culture in terms of like punctuality, being on time to places, the amazing public transport system they have, […], I’ve grown to enjoy*. (B01)

This more positive attitude helped me to get into the masters, since I had been worried about doing rotations. *It’s a very like a village-like hospital with a lot of local people who speak dialect, […] and have not probably seen a lot of people of color or immigrants […]*. (B01). So I worried. *How are the patients gonna look at me?* (B06)

Overall, my worries proved to be invalid, except on certain occasions. *I remember there was a case about a patient who was from [Brazil], and the specialist was talking about how obese she was […] they were making jokes about the fact that she is very diabetic and how her kidneys were probably damaged, and therefore she can get her kidneys off the black market because it’s so common there. And it took me a while to understand what this comment meant. And of course everyone was laughing and I was the only person who happened to be near that region […] I just felt very uncomfortable. […], definitely didn’t feel like I belonged* (B01).

I am in my final year now and proud of what I have achieved: by working hard, developing a tough skin, looking for likeminded peers outside university, and becoming more aware of my talents and challenges, I pushed through. I am confident to continue my medical career, but definitely outside European Netherlands.

### Student 3: Gladiolus

The selection process for the English programme was long and tough. *I had a typical* [Pakistani] *diploma […]. And that was like a whole process to get that accredited by the government*. (B06)

*I already knew I was up to the level, but like just* them *recognizing that. So I was happy about that* [*…*]. *The fact that just my classroom was full of white people that got me so excited […]. And I think that’s kind of what I was so looking forward to*. (B06)

But I felt completely lost. *If you just look at people in the lecture hall, you kind of do stand out. […] For example, when they [….] were talking about [Asia] ‘[…]one student asked me like ‘hey, is it true that hospitals are really bad over there?’ […] I think it’s more out […] of ignorance or something*. (B02)

*I don’t see [diversity] in my […] study. A lot of the diseases that we have are based on the Caucasians*. (B16) Many teachers and peers disrespect healthcare outside The Netherlands. *[…] I just act like I didn’t hear it* (B02). I do not show that I am feeling very lonely.

Before coming to Maastricht, in the conservative province of Limburg, I was a little scared: *the entire LGBT thing for me is also very important because I’m part of that community and I know how some people can be when they even think that you can be part of that community* (B15).

During the rotations, *for example with the GP rotations, we’re placed in areas of town where, […] people from low socioeconomic classes lived and […] like how shocked they*, my peers, *were about, […] their living standards, unhealthy behaviors like smoking and drug abuse and alcohol abuse. […] Yeah again this kind of ignorance* [among my peers] (B12).

*I also saw a lot of* [Pakistani] *patients, […] it was fun asking where are you from, […] you still create a bond. (B12)* While many peers find these patients difficult to communicate with, I find it an enjoyable challenge trying to understand and help these patients, to whom I can more easily relate because of my background.

This was an exceptional positive experience. After many years of struggles and hard work, I am heavily disappointed and frustrated, and I seriously consider quitting my studies.

### Reflections guided by theory

#### Multiple dynamic categories and experiences

Students Dahlia, Crocus and Gladiolus had multiple experiences, which they sometimes related to belonging to a specific group or category, but not always. For instance, in the selection process, Crocus and Gladiolus linked experiences to them being Dutch or Pakistani, whereas Dahlia described the process as smooth without reference to personal background. All three mentioned overlapping categories such as nationality and skin colour, but the categories mentioned also differed: Dahlia highlighted religion and age, Crocus emphasised gender and ethnicity, while Gladiolus focused on sexual orientation and ethnicity.

#### Belonging and exclusion

Students connected certain (but not the same) categories to feelings of belonging, such as religion, being in the English programme, ethnicity, LGBT identity, or regional affiliation. At the same time, categories were mentioned to create boundaries between groups, for example Dutch versus English programme, male versus female, black versus white, or Dutch versus non-Dutch speaking. Each student described experiences of exclusion: Dahlia through religion, Crocus through sex and ethnicity, and Gladiolus through skin colour and ethnicity.

#### Avowed and ascribed identities

Students categorised themselves, they labelled others and described how they think others labelled them. This labelling by and of others could coincide with students’ labelling of themselves (based on for ex. shared religion or international experience), but they also felt categorised in ways that did not align with their self-perceptions. For example, students felt peers and supervisors sometimes overlooked their ethnicity or socioeconomic background. Uncertainty about how they might be perceived (for ex. by conservative community or patients in rural hospital) produced insecurity.

#### Dynamic meanings of categories

The meaning and appreciation attached to categories differed and changed depending on student, time, and context (application procedure, the lecture hall, tutorial group meeting, the curriculum contents, patient interactions, and hospital environment). Crocus initially felt isolated as the only black student, while Gladiolus embraced the same circumstance. Nationality eased Crocus’s admission but did not prevent culture shock. Dahlia’s negative views of Dutch peers evolved positively over time. Gladiolus – unlike their peers – appreciated interacting with foreign patients and felt sympathetic to patients from low socioeconomic status. Categories thus carried both positive and negative meanings, with different connotations (fast/slow learners, racist terminology, jokes about patients’ ethnicity).

#### Deviating from implicit norms

Students stated how categorisation has different effects. They encountered stereotyping (e.g. being religious perceived by colleagues as being ‘a big dumb’; being forced to represent an entire continent; stigma that healthcare in the Netherlands is better than elsewhere), not being sufficiently recognised for who they are (others not acknowledging the student being Black; lack of attention for non-Western patients in curriculum), and exclusion (making jokes of a patient with whom student shares ethnicity). Inequalities also emerged: white privilege, ease of admission with Dutch nationality, or assumptions of low performance for students from the Antilles. Coping strategies varied from silence and resilience to speaking out, seeking support networks, or even discontinuing studies.

#### Synthesis

Linking these reflections to the theories described in the introduction, we found medical students label themselves in multiple ways and as belonging to changing groups. We found medical students attach different meanings to these labels, depending on person, context, and time. We also found that tensions between avowed and ascribed labelling, and negative connotations of labels can lead to students feeling stereotyped and excluded. When such experiences are repeated, the effects may become serious, potentially leading to resignation. The interconnection of these elements makes categorisation a multilayered, dynamic phenomenon.

## Discussion

This study showed how categorization plays a role in the daily life of medical students. The insight that categorisation is a ubiquitous, multilayered, paradoxical, and dynamic process is not new. In health professions education, research on intersectionality, identity safety and (professional) identity formation has shown how students’ multiple, coexisting backgrounds influence their study experiences and professional identity formation (PIF) [25,36,37]; how (professional) identity formation is a complex, non-linear and fluid process and multifactorial phenomenon [38]; how social identity theory may help to examine group processes and enhance teamwork in healthcare [39], and that the use of intersectionality theory in medical education research can help unpack the complexities and interconnections of difference [40]. However, often such studies still take a selected set of categories as their starting point—an approach that, despite good intentions, risks reinforcing stereotypes and potentially distancing itself from its aim of inclusivity and equity. Additionally, the practical application of intersectional and identity theories often overlooks the true complexity and fluidity of categorisation. In a critical review of the conceptualisation of PIF, Mount et al. (2022) found that only one PIF intervention discussed intersectionality, with limited attention for contextual factors and potential power hierarchies underlying PIF experiences [41]. Moreover, Zanting et al. (2025) highlighted that applying ‘intersectionality’ may disregard how changing understandings of characteristics, contexts, and hierarchies play a role in people’s daily experiences [42]. While social identity theories pay attention to interrelated elements of categorisation, identity formation and comparison, our study adds insights by focussing on unpacking the element of categorisation. By examining medical students’ daily encounters with categorisation without predefined categories, our study makes visible the nuanced ways this multilayeredness affects students’ study experiences, not only during social and professional interactions but also in relation to admission procedures and curriculum contents.

Because categorisation is so complex, clear patterns are difficult to identify. Nonetheless, it seems that students mentioned categories when they felt deviant from an implicit standard. In this specific context – a medical school in the Netherlands – the implicit standard appeared to be being white, Dutch, female, non-religious and heterosexual. No student mentioned being heterosexual, non-religious, or from a high socioeconomic background. The student who noted their whiteness was conscious of the associated privileges. The students who referred to their Dutch nationality either studied in the English programme surrounded by other nationalities, or came from the Dutch Antilles, and felt “different” within the Dutch program due to linguistic or cultural differences. So, in both cases, the students felt reason to perceive themselves as ‘non-standard’. As earlier studies confirm, what constitutes the implicit standard is noticeable mainly to those who do not belong to it, and oblivious to those who do [36,43]. Moreover, this standard is contextually constructed, not fixed.

Furthermore, it seems that when categories are similarly perceived by all people involved and labels are valued positively, students experience belonging. But when labels carry (implied) negative connotations, when students feel a category is overly emphasised, or neglected while found meaningful, the multidirectional categorisation can cause tension. Apparently, students’ learning experience is influenced by how categories are valued and whether the multidirectional categorisation aligns across persons and contexts. Some forms of categorisation have more serious impact, depending on context and individual. Although students did not explicitly mention systemic discrimination, the uncritiqued use of discriminatory terminology and a Caucasian-focused curriculum can be considered as a sign of systemic discrimination operating through everyday unconscious categorisation and built-in bias, which is rarely recognized [44]. It seems that when categories deviate from an implicit standard and carry negative connotations, and this stereotypical categorisation is repeatedly experienced, this situation seriously impacts students’ wellbeing and learning. These consequences are varied, and echoed by previous research documenting inequities in admissions [45], emotional distress and lack of belonging [4,6], biased curricula [46], discrimination [5], compromised identity safety [47], and potential reduced performance [11]. Our findings emphasise that such implications result from a complex interplay of categorisation dynamics, rather than from fixed ‘membership’ to certain categories.

Categories act as fluid occasional labels that help students make sense of themselves, others, and their daily experiences. Because categorisation is dynamic and multilayered, it is impossible to feel fully included at all times—occasional exclusion is part of human experience. Constructing categories, which is distinct from identifying characteristics, often arises as a way to make sense of uncomfortable interactions [16]. Our study suggests that the unpredictability of how others categorise them – when, how and with what meaning – creates insecurity, confusion, disappointment, and additional efforts for students. Currently, coping with this unpredictability seems left largely to the individual student.

Our findings suggest that institutions should not leave students to navigate these challenges alone. We recommend paying more attention to the existence and impact of implicit norms in all stages of educational practice: in selection criteria and processes, in biomedical curriculum contents, as part of assessment practices and during classroom and supervisory interactions. Consistent with prior recommendations, our study supports calls to integrate students’ and patients’ personal experiences and contexts into PIF interventions [41], selection processes [48] and course materials [49], instead of focusing on standard groups or categories. Our study adds that feelings of belonging and exclusion are omnipresent and dynamic; raising awareness of this finding in all stages of education is a prerequisite for developing tangible tools to reduce inequity and enhance learning.

Similarly, research design should move beyond focusing on predefined categories toward exploring individual experiences and implicit norms. This approach prevents categories from becoming overly rigid and helps ensure that attention is not limited to a narrow set of categories. A more open and flexible approach to identifying relevant differences and investigating the implicit norms in specific contexts is recommended.

Although the Dutch setting shaped which categories surfaced in this study, the underlying processes of categorisation are likely comparable across contexts. Our data represent students’ perspectives on categorisation—including how others categorised them—but not the perspectives of those “others.” To expand on insights from this initial exploration of the impact of categorisation in educational practice, and to overcome risks pertaining to staying uninformed about this impact, it is suggested that future research examines categorisation across varied settings and stakeholders, and explores its role in specific aspects of medical education, such as admissions, curriculum design, classroom interactions or clinical rotations.

## Conclusion

This study suggests that students’ learning experiences can be improved by recognising the omnipresent and dynamic nature of categorisation. Developing conscious awareness of relevant and nuanced differences—and focusing on individuals’ experiences and implicit norms instead of on standard categories in both educational practice and research design—is crucial for fostering and sustaining a diverse student body, an equitable learning environment, and ultimately a more inclusive healthcare workforce.
